# Application of 3D-QSAR, Pharmacophore, and Molecular Docking in the Molecular Design of Diarylpyrimidine Derivatives as HIV-1 Nonnucleoside Reverse Transcriptase Inhibitors

**DOI:** 10.3390/ijms19051436

**Published:** 2018-05-11

**Authors:** Genyan Liu, Wenjie Wang, Youlan Wan, Xiulian Ju, Shuangxi Gu

**Affiliations:** Key Laboratory for Green Chemical Process of Ministry of Education, School of Chemical Engineering and Pharmacy, Wuhan Institute of Technology, Wuhan 430205, China; wangwenjie0610@gmail.com (W.W.); wanyoulan1994@gmail.com (Y.W.); xiulianju2008@aliyun.com (X.J.)

**Keywords:** HIV-1 NNRTIs, CoMFA, CoMSIA, pharmacophore, docking, molecular design

## Abstract

Diarylpyrimidines (DAPYs), acting as HIV-1 nonnucleoside reverse transcriptase inhibitors (NNRTIs), have been considered to be one of the most potent drug families in the fight against acquired immunodeficiency syndrome (AIDS). To better understand the structural requirements of HIV-1 NNRTIs, three-dimensional quantitative structure–activity relationship (3D-QSAR), pharmacophore, and molecular docking studies were performed on 52 DAPY analogues that were synthesized in our previous studies. The internal and external validation parameters indicated that the generated 3D-QSAR models, including comparative molecular field analysis (CoMFA, $q^{2}$ = 0.679, $R^{2}$ = 0.983, and $r_{\mathrm{pred}}^{2}$ = 0.884) and comparative molecular similarity indices analysis (CoMSIA, $q^{2}$ = 0.734, $R^{2}$ = 0.985, and $r_{\mathrm{pred}}^{2}$ = 0.891), exhibited good predictive abilities and significant statistical reliability. The docking results demonstrated that the phenyl ring at the C_4_-position of the pyrimidine ring was better than the cycloalkanes for the activity, as the phenyl group was able to participate in π–π stacking interactions with the aromatic residues of the binding site, whereas the cycloalkanes were not. The pharmacophore model and 3D-QSAR contour maps provided significant insights into the key structural features of DAPYs that were responsible for the activity. On the basis of the obtained information, a series of novel DAPY analogues of HIV-1 NNRTIs with potentially higher predicted activity was designed. This work might provide useful information for guiding the rational design of potential HIV-1 NNRTI DAPYs.

## 1. Introduction

Acquired immunodeficiency syndrome (AIDS) is a potentially fatal infectious disease caused by the human immunodeficiency virus (HIV) [1]. The lack of effective vaccines or drugs at present is still a major obstacle in the fight against HIV infection [2,3]. According to the data reported by the World Health Organization on 17 May 2017, it is estimated that approximately 1.1 million people died from HIV-related diseases in 2015, and 36.7 million people had been infected with HIV by the end of 2015. By mid-2016, 18.2 million AIDS patients worldwide were administered the highly active antiretroviral therapy (HAART), which can dramatically reduce the mortality of HIV-infected patients by inhibiting HIV replication [4].

**Figure 1:**
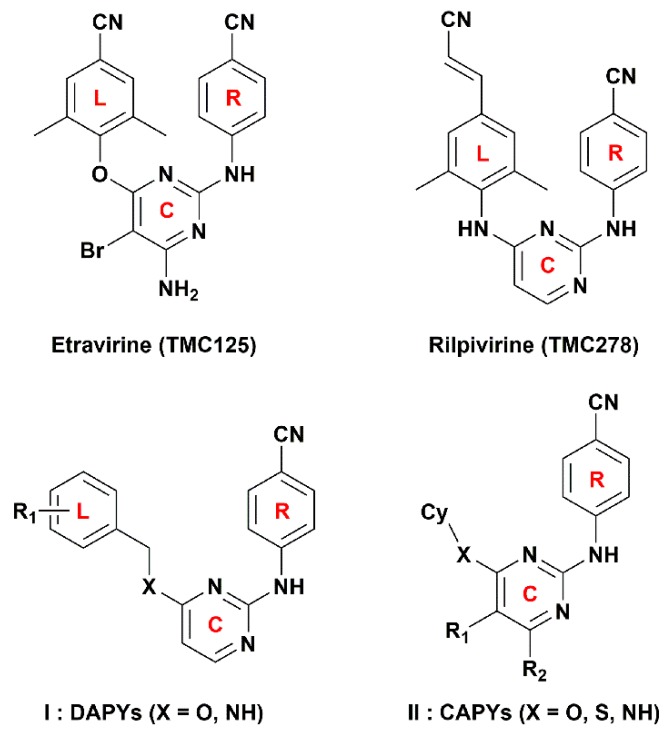
Structures of diarylpyrimidines (DAPYs) and cycloalkyl arylpyrimidines (CAPYs). The DAPYs scaffold includes a left wing (L), a right wing (R), a central pyrimidine ring (C), and the linkers.

Nonnucleoside reverse transcriptase inhibitors (NNRTIs), as an indispensable part of HAART, have attracted wide attention because of their potent antiviral activity, high specificity, and low cytotoxicity [5]. The NNRTIs mainly inhibit the reverse transcriptase (RT) of HIV type 1 (HIV-1) by binding to a hydrophobic pocket localized about 10 Å from the catalytic site of the enzyme [6,7]. Until now, a large amount of NNRTIs with diverse chemical structures have been reported, such as dihydroalkoxybenzyloxopyrimidines, benzophenones, diaryl ethers, diaryltriazines, and diarylpyrimidines (DAPYs). Among these series of NNRTIs, DAPYs are considered to be one of the most successful anti-HIV families [8]. Etravirine (TMC125) and rilpivirine (TMC278) (Figure 1), two representative members of DAPYs, have been approved by the U.S. Food and Drug Administration in 2008 and 2011, respectively. Compared to the first or second generation of NNRTIs, such as delavirdine and efavirenz, etravirine and rilpivirine exhibit excellent potencies against wild-type (WT) HIV-1 and resistant mutants such as K103N. However, hypersensitivity reactions, rash, and Stevens–Johnson syndrome were observed in clinical cases of etravirine [9]. Additionally, AIDS patients in which therapy failed were found to show increased drug resistance to rilpivirine compared to efavirenz [10].

Because of the fact that the right wing of the DAPY structure has been confirmed as a key pharmacophore for anti-HIV-1 activity [11], the structural modifications were mainly carried on the left wing, the central pyrimidine ring, and their linker (L–C bridge) (Figure 1). Recently, we synthesized a series of DAPYs (Figure 1) by modifying the left wing as well as the linker, and most of them exhibited potent anti-HIV-1 activity [5,8]. However, when modifying the left wing, the modifications were often confined to different substituted aromatic groups without considering cycloalkyl groups. Thus, in a previous study, we also examined whether the introduction of a cycloalkyl group on the left wing of DAPYs to replace the phenyl group could provide a novel structural scaffold to improve the anti-HIV-1 activity [11]. Several synthesized cycloalkyl arylpyrimidines (CAPYs) (Figure 1) also show moderate anti-HIV-1 activity. However, the three-dimensional quantitative structure–activity relationships (3D-QSARs) of these DAPYs and CAPYs and their interaction mechanisms as HIV-1 NNRTIs were not well understood.

To further explore the relationships between the inhibitory activity of HIV-1 NNRTIs and their structural features, in this work, 3D-QSAR studies including comparative molecular field analysis (CoMFA) and comparative molecular similarity indices analysis (CoMSIA) were applied on a series of DAPYs and CAPYs. In addition, pharmacophore modeling and molecular docking were performed to investigate the binding pattern of DAPYs and CAPYs with the enzyme. All the developed models could provide some useful information about structural modifications in designing novel and potent DAPYs as HIV-1 NNRTIs.

## 2. Results and Discussion

### 2.1. CoMFA and CoMSIA Statistical Results

The classical parameters of CoMFA and CoMSIA models, including $q^{2}$, *ONC*, $R^{2}$, $r_{\mathrm{pred}}^{2}$, *SEE*, and *F*-values, are listed in Table 1. The other important validation parameters, such as *RMSE*, *MAE*, $r^{2}$, $r_{0}^{2}$, $r_{0}^{\prime 2}$, *k*, *k’*, $r_{m}^{2}$, $r_{m}^{/2}$, Δ$r_{m}^{2}$, and $\bar{r_{m}^{2}}$ are listed in Table 2. The results from the CoMFA model indicated that $q^{2}$, $R^{2}$, $r_{\mathrm{pred}}^{2}$, *MAE*, *RMSE*, Δ$r_{m}^{2}$, and $\bar{r_{m}^{2}}$ were 0.679, 0.983, 0.884, 0.124, 0.160, 0.1215 (or 0.0026), and 0.7829 (or 0.9690), respectively. These data proved that the constructed CoMFA model was reliable, and its predictive accuracy was acceptable ($r_{\mathrm{pred}}^{2}$ > 0.5). The steric and electrostatic fields contributions were 46.30% and 53.70%, respectively, indicating that the electrostatic fields gave an important contribution.

For the CoMSIA analysis, different combinations of descriptor fields were used to construct different CoMSIA models. All possible combinations of fields were performed to determine the optimal predictive model [12]. According to the experimental data in Table 1, it could be found that a model consisting of steric, electrostatic, hydrophobic, hydrogen-bond donor and hydrogen-bond acceptor fields led to relatively higher $q^{2}$, $R^{2}$, $r_{\mathrm{pred}}^{2}$ and relatively lower *SEE*. Therefore, the model (S + E + H + D + A, Table 1) was considered to be the best possible combination, which assigned satisfactory values to the parameters $q^{2}$, $R^{2}$, $r_{\mathrm{pred}}^{2}$, *MAE*, *RMSE*, Δ$r_{m}^{2}$, and $\bar{r_{m}^{2}}$, i.e., 0.734, 0.985, 0.891, 0.108, 0.155, 0.1387 (or 0.0171), and 0.7389 (or 0.9763), respectively. The corresponding contributions of steric, electrostatic, hydrophobic, hydrogen-bond acceptor, and hydrogen-bond donor fields were 12.30%, 41.40%, 27.60%, 7.5%, and 11.20%, respectively. Compared to the CoMFA model, the CoMSIA model seemed to show a somewhat better predictivity. It was also found that the electrostatic fields were significant contributors in the optimal CoMSIA model. These results indicate that the constructed models are powerful to predict the activity of DAPYs and CAPYs. The field contributions revealed that the electrostatic fields play important roles in the CoMFA and CoMSIA models.

**Figure 2:**
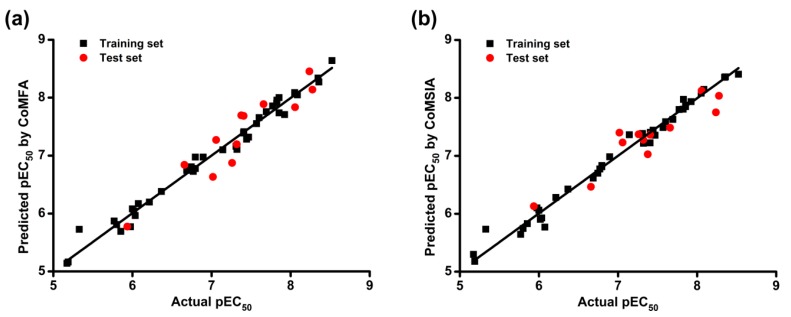
Scatter pot of actual against predicted pEC_50_ values. (**a**) CoMFA model; (**b**) CoMSIA model.

Then, we used the models to predict the activity of the training and test set compounds. The actual and predicted pEC_50_ (−logEC_50_) values of DAPYs and CAPYs are listed in Table 3. The correlations between actual and predicted pEC_50_ values are shown in Figure 2. The predicted pEC_50_ values were close to the experimental data, and most of the points were located on or near the trend line, which indicated the predictivity and reliability of both models. The above results indicate that constructed CoMFA and CoMSIA models are reasonable and have the ability to predict the anti-HIV-1 activity of the training and test compounds of DAPYs and CAPYs.

### 2.2. CoMFA and CoMSIA Contour Maps

To visualize the different field effects in three-dimensional spaces where the modifications could increase the activity of the target compounds, contour maps were generated subsequently in the CoMFA and CoMSIA models. Compound **43** with the highest activity was used as a reference structure to illustrate all the contour maps.

**Figure 3:**
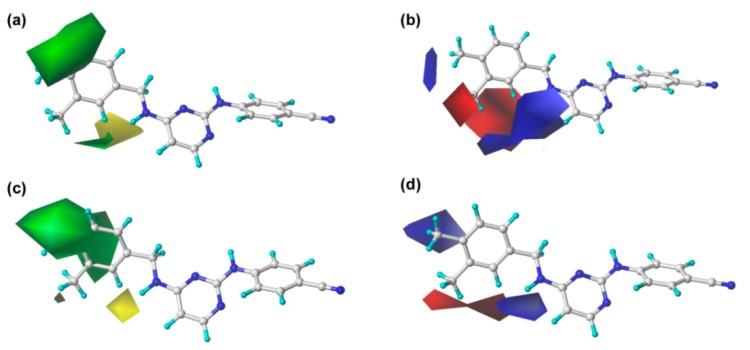
The contour maps of the steric and electrostatic fields in combination with compound **43**. Steric fields: favored (green) and disfavored (yellow). Electrostatic fields: electropositive (blue) and electronegative (red). (**a**,**b**) CoMFA model; (**c**,**d**) CoMSIA model.

The steric and electrostatic contour maps of CoMFA and CoMSIA are shown in Figure 3. In the steric fields, the green contours indicate sterically favorable bulky substituents, whereas the yellow contours indicate where the substituents are sterically unfavorable [13]. It should be noted that the steric contour maps of CoMSIA are similar to those of the CoMFA model, which proves the consistency of the results. As shown in Figure 3, a large green contour surrounding the C_3_ (or C_5_) and C_4_ positions of the left phenyl ring indicates that bulky groups here are beneficial for enhancing the activity. This finding might explain why the activity of compound **43** is significantly higher than that of other compounds. However, the structure–activity relationships of several compounds with single substitution at the C_3_ or C_4_ position were not coincident with this finding, as seen, for example, for compounds **6** (4-Br) < **9** (4-F). This might have been caused by other properties of the substitutions or the effects of other fields and remains to be studied. On the other hand, there was a small yellow contour surrounding the C_2_-position of the left phenyl ring, which suggested that bulky substituents at this site might be unfavorable for the activity, as observed for the following compounds in this order: **4** (2-Br) < **1** (2-Cl) < **7** (2-F) and **16** (2-CF_3_) < **10** (2-CH_3_) < **24** (2-H).

For the electrostatic maps, the blue contours denote regions where positively charged substituents will improve the inhibitory activity, whereas the red regions show negatively charged substituents are helpful for enhancing the activity. As shown in Figure 3, a large blue contour around the C_4_-position of the left phenyl ring reveals that the positively charged substituents at this position are favorable for increasing the inhibitory activity. This finding can be supported by examples as follows: compound **36** with a methyl substituent at the C_4_-position showed higher inhibition activity compared to compounds **30** (4-Cl), **33** (4-Br), and **35** (4-F); the activity order was **36** (4-CH_3_) > **33** (4-Br) > **30** (4-Cl) > **35** (4-F). Moreover, a big blue irregular contour near the linker between the left wing and the central pyrimidine indicates that the presence of a positively charged group in this region is beneficial to the bioactivity, which is consistent with the experimental data, showing, for example, **27** (linker = –NH) > **24** (linker = –O). Two small red contours surrounding the C_2_ and C_3_ positions of the left phenyl ring, respectively, indicating that the presence of negative charges in these regions will be favorable for bioactivity. For example, compounds **1** (2-Cl), **4** (2-Br), and **7** (2-F) showed higher activity than compound **10** (2-CH_3_), and the order of their inhibitory activity was **7** (2-F) > **1** (2-Cl) > **4** (2-Br) > **10** (2-CH_3_). This result is also reflected in the fact that the activity of compound **34,** with a fluorine (negative charge) at the C_3_-position, was increased significantly compared with that of compound **41** bearing a trifluoromethyl (positive charge).

**Figure 4:**
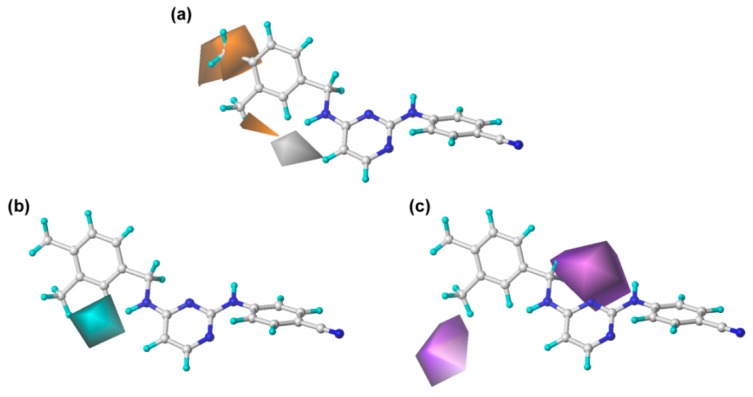
The CoMSIA contour maps in combination with compound **43**. (**a**) Hydrophobic fields: hydrophobic (orange) and hydrophilic (white). (**b**) Hydrogen-bond donor fields: favored (cyan) and disfavored (purple, not present). (**c**) Hydrogen-bond acceptor fields: favored (green, not present) and disfavored (violet).

As shown in Table 1 and Figure 4a, the hydrophobic field also plays an important role in the optimal CoMSIA model. Two large orange contours are located near the C_3_ and C_4_ positions of the left phenyl ring, indicating that a hydrophobic substituent in these two positions will be favorable to the inhibitory activity. For example, compound **43** with methyl groups in these two zones showed higher activity than compound **24** with hydrogens. Besides, there is a white contour close to the C_2_-position of the left phenyl ring, which suggests that the presence of a hydrophilic group in this position might increase the inhibitory activity. For instance, the activity of compound **7** with a fluorine group at the C_2_-position of the left phenyl ring was higher than that of compound **24** with a hydrogen at this position.

Furthermore, the hydrogen-bond donor and acceptor fields also play relatively important roles in the bioactivity of the compounds. As shown in Figure 4b, c, a cyan contour surrounds the linker between the central pyrimidine and the left wing, which signifies that hydrogen-bond donor groups at this position will be beneficial to the bioactivity. This can be certified by the fact that the inhibitory activity of compound **27** (linker = –NH) was significantly higher than that of compound **24** (linker = –O). Moreover, it also can be observed that there are two violet contours around the C_3_-position of the left phenyl ring and the X substituent of the linker (Figure 1), respectively, indicating that hydrogen-bond acceptor groups in these positions are not beneficial for the biological activity. This result is supported by the biological activity of the compounds that contain an oxygen atom as X substituent, such as compounds **45**, **46**, and **47**.

### 2.3. Pharmacophore Model

**Figure 5:**
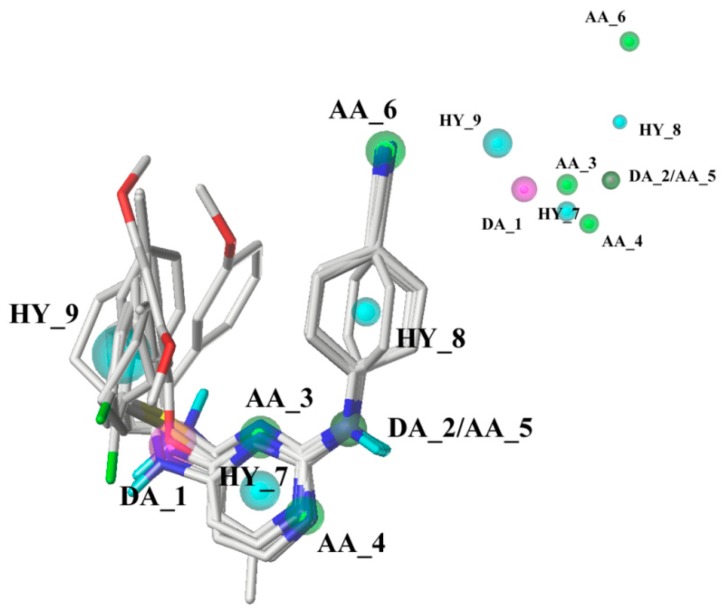
The pharmacophore model with the alignment of nine training set compounds. The magenta, green, and cyan spheres represent hydrogen-bond donor atoms (DAs), hydrogen-bond acceptor atoms (AAs), and hydrophobes (HYs), respectively.

The pharmacophore model was constructed using nine compounds with diverse structures and relatively high activities as a training set. Twenty pharmacophore models were generated after Genetic Algorithm with Linear Assignment of Hypermolecular Alignment of Datasets (GALAHAD) run, each of which represented a different trade-off among the competing criteria. The lower the strain energy (SE) values, and the higher the steric overlap (SO) and pharmacophoric similarity (PhS) values, the better the model. According to the experimental results, it was found that the parameters of the best generated model were: SE = 2.982, SO = 255.80, and PhS = 123.30. The pharmacophore model with the alignment of nine compounds is shown in Figure 5, indicating a satisfactory superimposition. As depicted in Figure 5, the magenta, green, and cyan spheres represent hydrogen-bond donor atoms (DAs), hydrogen-bond acceptor atoms (AAs), and hydrophobes (HYs), respectively. The best model is formed by nine pharmacophore features: two hydrogen-bond DAs, four hydrogen-bond AAs, and three HY centers. One of the hydrogen-bond DAs is the nitrogen atom of an imine group, and the four hydrogen-bond AAs correspond to the nitrogen atoms of a pyrimidine ring, imine group, and nitrile group, respectively. These features reflect the importance of the DAPY/CAPY common scaffold for the inhibitory activity. Another hydrogen-bond DA is located at the linker atom, indicating that a hydrogen-bond donor groups such as –NH at this position may increase the inhibitory activity, which is in accordance with the hydrogen-bond donor fields results in the CoMSIA contour maps. The three hydrophobic centers are located at the center of the left phenyl ring, the center of the pyrimidine ring, and the center of the right phenyl ring, respectively, which suggests that a large hydrophobic structure on the left wing is favorable for the activity. These results are in agreement with the actual activities and the steric fields of the 3D-QSAR contour maps.

### 2.4. Molecular Docking Analysis

**Figure 6:**
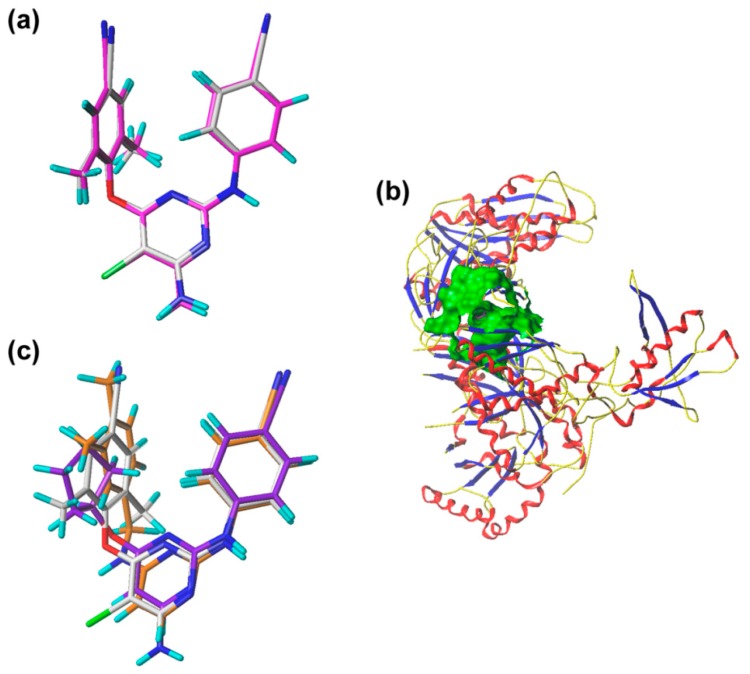
The superimposition of docked compounds and binding pocket. (**a**) The superimposition of the cognate etravirine and the redocked etravirine. The magenta sticks represent the cognate etravirine, and the white sticks represent the redocked etravirine. (**b**) The green region represents the surface of the binding pocket. (**c**) Superimposition of compounds **43**, **46**, and redocked etravirine. The orange sticks represent the compound **43** with highest activity; the purple sticks represent the compound **46** with lowest activity; the white sticks represent redocked etravirine.

To validate the docking reliability, a cognate ligand, i.e., etravirine, which was extracted from the crystal structure of the WT HIV-1 RT (PDB ID: 3MEC), was first re-docked into the binding site using surflex-docking. The redocked conformation was compared with the original crystallographic conformation of the ligand [14]. As shown in Figure 6a, the redocked etravirine and the crystal etravirine in the complex are almost completely superimposable, and the root-mean-square deviation (RMSD) of the two conformations for all atoms is 0.25 Å. These results suggest that the surflex docking method and the used parameters are reasonable and reliable [15]. The DAPYs and CAPYs were then docked into the binding site in the same way. The generated binding pocket is shown in Figure 6b.

After validating the docking reliability, all the DAPYs and CAPYs were docked into the binding pocket. The superimposition of the most active compound **43** and the least active compound **46** with the redocked etravirine is shown in Figure 6c. It should be noted that compound **43** superimposes with etravirine better than compound **46,** although their docking conformations are in a similar orientation. The docking score of compound **43** (total-score = 9.5247) was higher than that of compound **46** (total-score = 8.2434), which is in agreement with their activities.

**Figure 7:**
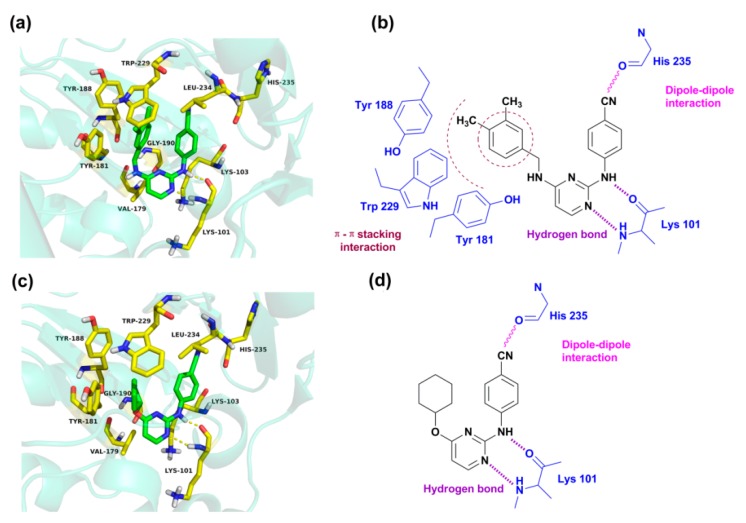
Docking results of compounds **43** (**a**) and **46** (**c**) in the nonnucleoside binding site of human immunodeficiency virus type 1 (HIV-1) reverse transcriptase (RT). Hydrogen bonds are shown as yellow lines, and compounds **43** and **46** are represented as green sticks. The schematic diagram of the interacting modes for compound **43** (**b**) and **46** (**d**), respectively.

Figure 7 presents the detailed interacting modes of compounds **43** and **46** in the binding site of the HIV-1 RT (3MEC). As seen from Figure 7a,c, the two compounds have the same orientation and adopt a horseshoe or a “U”-shaped conformation in the pocket, as previously reported [16,17]. As shown in Figure 7a,b, the backbone of Lys 101 forms two hydrogen bonds with the nitrogen atoms of the pyrimidine and -NH linker of compound **43**, respectively. This result is in agreement with our previous report that the residue Lys101 might interact with DAPYs and CAPYs through hydrogen bonds [8]. The same interactions were also observed in the binding mode of compound **46**. The hydrogen bond distances and angles are shown in Table 4.

It was also found that some amino acid residues in the binding pocket, including Tyr318, Tyr232, Phe 227, Trp239, Trp229, Pro225, Pro226, Met230, Ile94, and Val189, formed hydrophobic interactions with compounds **43** and **46** [18]. According to the pharmacophore model, it could also be concluded that bulky lipophilic substituents, such as an aromatic ring on the left wing of DAPYs, might make hydrophobic contacts with these amino acid residues. Moreover, van der Waals interactions could be established between the docked compounds and amino acid residues such as Leu100, Lys103, Val179, Gly190, and Leu234. The cyano group in the right aryl wing could establish a dipole–dipole interaction with the carbonyl of His235. These interactions might allow the inhibitors to maintain a horseshoe or a “U”-shaped conformation.

Additionally, π–π stacking interactions were found between the left phenyl ring of compound **43** and aromatic amino acid residues such as Tyr188, Tyr181, and Trp229 [11,19]. As shown in Figure 7a, the left phenyl group is parallel to Tyr181 or Tyr188, and the 4-CH_3_ on the phenyl ring points towards the highly conserved Trp229. However, π–π stacking interactions are not found in the docking results of compound **46** because of its lack of an aromatic ring on the left wing. The results indicate that the cyclohexyl or cyclopentyl substituents on the left wing of CAPYs are unfavorable for inhibitory activity, which might be due to the loss of the π–π stacking interactions.

### 2.5. Newly Designed DAPYs

Based on the combination analysis of the 3D-QSAR, pharmacophore, and molecular docking results, structure–activity relationships of DAPYs were obtained and subsequently utilized to design new DAPYs as potential HIV-1 NNRTIs. Ten novel DAPYs were designed, and their anti-HIV-1 activities were predicted by the CoMFA and the best CoMSIA models, as seen in Table 5. Several principles were considered in the design of these novel DAPYs. First, the left phenyl ring was retained as a core moiety in the designed compounds because it is able to participate in π–π stacking interactions with the aromatic amino acid residues in the binding pocket. Second, the contour maps of the hydrogen-bond donor fields and pharmacophore features indicate that hydrogen-bond donors located at the left linker are preferred to enhance the activity, thus an imino group was retained as a linker instead of an oxygen atom. Third, different substitutions were introduced into the left phenyl group according to the contour maps analysis as follows: (a) a bulky, positively charged, and/or hydrophobic substituent, such as –CH_2_CH_3_, –CH(CH_3_)_2_, –C(CH_3_)_3_, and –NH_2_, at the C_4_-position; (b) a negatively charged and/or hydrophobic group, such as –CN, –NO_2_, and –OOCCH_3_, at the C_3_-position; (c) a small, negatively charged, and/or hydrophilic substituent, such as –OH and –F, at the C_2_-position.

As shown in Table 5, the predicted activities of the newly designed molecules are all remarkable. Three compounds (**54**, **60**, and **62**) demonstrated a higher activity in the CoMFA and the optimal CoMSIA models than the most active compound **43**. These results indicate that the molecular simulation study was able to provide a reference for optimizing the structure and evaluating new potent DAPYs. However, future studies on synthesis methods, activity assays, and pharmacokinetic tests of these newly designed DAPYs are necessary.

## 3. Materials and Methods

### 3.1. Dataset and Alignment

**Figure 8:**
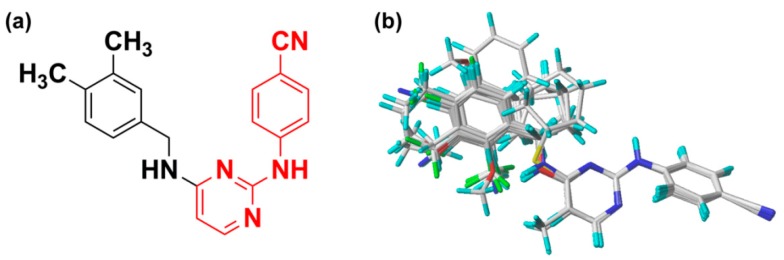
Compound **43** was used for the alignment of all molecules. (**a**) The structure of compound **43** corresponding to the common scaffold is in red; (**b**) Alignment of the training set compounds.

Fifty-two DAPY and CAPY compounds belonging to the HIV-1 NNRTI class were obtained from our previous studies and used to set up the 3D-QSAR models [5,8,11]. All the compounds were randomly divided into two sets, including 40 compounds as a training set to generate the model and 12 compounds as a test set to validate the model. The bioactivity of all the compounds was converted into −logEC_50_ (pEC_50_). Their structures and bioactivity are given in Table 3. All calculations were carried out using the SYBYL-X 2.1 software (Tripos Inc., St. Louis, MO, USA) running on a windows 7 workstation. Energy optimization of all molecules used the Gasteiger–Hückel charges and Tripos force field with a gradient descent method, with a gradient convergence criterion of 0.005 kcal/mol·Å and a maximum iteration coefficient of 10,000 [20]. The other parameters were set to default values. In order to obtain an optimal alignment, the conformation of compound **43** with the highest bioactivity was selected as a template, and the other molecules were aligned on it by common substructure alignment and manual adjustment. The common skeleton (red atoms) for the molecular alignment is shown in Figure 8a, and the superimposed structures of the training set are shown in Figure 8b.

### 3.2. CoMFA and CoMSIA Models

The 3D-QSAR models were generated using the CoMFA and CoMSIA methods which could help us better understand visually the relationships between the structural features of DAPYs and their inhibitory activity [21]. The alignment quality has an important impact on the robustness and predictivity of the generated models [22]. The contour maps of the CoMFA and CoMSIA models were graphically presented using the field type of “Stdev*Coeff”. For the CoMFA models, the physicochemical properties, such as the steric and electrostatic fields, were calculated at every grid point of a regularly grid spacing of 2.0 Å, using a sp^3^ hybridized carbon probe with a +1 charge. For the CoMSIA models, five physicochemical properties, including steric, electrostatic, hydrophobic, hydrogen-bond donor and acceptor fields, were respectively calculated by using the same lattice box that were also used in the CoMFA model and a sp^3^ carbon probe with +1 charge, +1 hydrophobicity, +1 hydrogen-bond donor, and +1 hydrogen-bond acceptor properties.

To verify the reliability of the generated models, internal validation and external validation are usually performed [23]. The leave-one-out (LOO) method is used for the internal validation and extracts a molecule from the dataset as a test set and considers the rest of the molecules as a training set to generate the QSAR models and predict the extracted molecule. This methodology can gain the optimal number of components (*ONC*) based on the highest cross-validated correlation coefficient ($q^{2}$) [23,24], which is defined as follows:

(1) $$q^{2}=1-\frac{\sum {{(r}_{\mathrm{pred}}-r_{\exp})}^{2}}{\sum {{(r}_{\exp}-r_{\mathrm{mean}})}^{2}}$$

where r_pred_, r_exp_, and r_mean_ represent the predicted, experimental, and mean pEC_50_ values, respectively.

For the external validation, the predictive correlation coefficient ($r_{\mathrm{pred}}^{2}$) is usually used to judge the quality of prediction, which can be calculated according to the following formula:

(2) $$r_{\mathrm{pred}}^{2}=\frac{\mathrm{SDEP}-\mathrm{PRESS}}{\mathrm{SDEP}}$$

where the standard deviation error of predictions (*SDEP*) is the sum of the squared deviations between the experimental activities of the test set and the mean activities of the training set compounds, and the predicted residuals sum of squares (*PRESS*) is the sum of the squared deviations between the predicted and the experimental activity for all compounds of the test set.

In addition, a novel metric $r_{m}^{2}$, is also used for additional internal validation (training set validation) and external validation (test set validation) represented by $r_{m(\mathrm{LOO})}^{2}$ and $r_{m(\mathrm{test})}^{2}$, respectively. This metric can be calculated according to the following equation:

(3) $$r_{m}^{2}=r^{2}\times (1-\sqrt{r^{2}-r_{0}^{2}})$$

where $r^{2}$ and $r_{0}^{2}$ are determination coefficients for the least squares regression with and without intercept, respectively, which is based on predicted pEC_50_ values in the *x*-axis and experimental pEC_50_ values in the *y*-axis.

In order to avoid the overestimation of the predicted quality resulting from classical metrics ($q^{2}$ and $r_{\mathrm{pred}}^{2}$), several other important validation parameters such as *RMSE* (root mean square error), *MAE* (mean absolute error) [25], *k*, *k*’, and different $r_{m}^{2}$ values ($r_{m}^{/2}$, Δ$r_{m}^{2}$ and $\bar{r_{m}^{2}}$) are also used to estimate the quality of the predictions. A model is eligible to be selected for further analysis if the following requirements are satisfied: $q^{2}$ > 0.5, $R^{2}$ > 0.6, $r_{\mathrm{pred}}^{2}$ > 0.5, 0.85 ≤ *k* (or *k’*) ≤ 1.15, $\frac{r^{2\;}-{\;r}_{0\;}^{2}(r_{0}^{\prime 2})}{r^{2}}<0.1$, Δ$r_{m}^{2}$ < 0.2 and $\bar{r_{m}^{2}}$ > 0.5 [23,25,26].

### 3.3. Pharmacophore Model

The pharmacophore hypothesis model was generated using the Tripos proprietary technology, GALAHAD, which is capable of identifying a set of ligand conformations of the optimal combination and is mainly determined by strain energy (SE), steric overlap (SO), and pharmacophoric similarity (PhS) [27].

To avoid redundancy of information, similar compounds with similar affinities were removed. Nine molecules were chosen to develop the pharmacophore model on the basis of their high activities and diverse structures. These compounds were aligned with each other, with a population size of 50 and a maximum generation of 100. Then GALAHAD parameters were set to Hypermolecule Construction. Default values were used for the other settings. According to the Pareto ranking, the model with the best SE, SO, and PhS values was chosen for analysis.

### 3.4. Molecular Docking

It is well known that molecular docking is of great significance to understand the mechanisms of interaction between the ligand and the receptor protein when designing new chemical molecules. In order to analyze the intermolecular interactions between DAPYs/CAPYs and HIV-1 RT effectively, molecular docking was implemented using the surflex-docking package of Sybyl-X 2.1. A cocrystal of WT HIV-1 RT with TMC125 (3MEC), whose ligand is highly similar to DAPYs, was obtained from RCSB Protein Data Bank. Before docking, 3MEC was prepared by removing water and sulfate ions and extracting the ligand [28]. Besides that, addition of hydrogen and charges and treatment of the terminal residues were also performed on 3MEC. Then, the “protomol” was generated by adopting the ligand-based mode, and an appropriate binding pocket was formed [29]. The reliability validation of the surflex-docking was conducted by redocking the cognate ligand into the binding pocket. Next, all of the DAPYs were docked into the binding pocket, and 20 possible docked conformations were obtained with different scores. Finally, the docked conformations of the most active compound **43** and the least active compound **46** were used to analyze the interaction mechanism.

## 4. Conclusions

In this paper, 3D-QSAR, pharmacophore, and docking studies were performed on 52 DAPY derivatives to investigate the relationships between their structures and anti-HIV activity. The CoMFA and CoMSIA models with high statistical significance and good predictive capacity were constructed, and a potential pharmacophore model was established. The docking results demonstrated the interaction modes of DAPYs in the binding pocket of the HIV-1 RT and suggested that the left phenyl ring of DAPYs played a key role in anti-HIV-1 activity. The pharmacophore model and 3D-QSAR contour maps allowed the visualization of the feature requirements to improve the activity. Several novel DAPYs with enhanced predicted activity were designed. However, these newly designed DAPYs remain to be synthesized and tested. Their pharmacokinetic profiles also need to be determined if they exhibit improved inhibitory activities against HIV-1 RT. Overall, the constructed models and obtained information have potential to be applied for further rational design of novel and potent DAPY analogues.

## Figures and Tables

**Table 1:** Classical statistical parameters of the comparative molecular field analysis (CoMFA) and comparative molecular similarity indices analysis (CoMSIA) models.

Model		$q^{2}$	*ONC*	$R^{2}$	$r_{\mathrm{pred}}^{2}$	*SEE*	*F*	Filed Contribution (%)				
								E	H	S	D	A
CoMFA	E + S	0.679	8	0.983	0.884	0.136	229.756	53.70	—	46.30	—	—
CoMSIA	E + H + S	0.721	9	0.972	0.734	0.180	114.912	52.40	32.40	15.20	—	—
	E + S + D + A	0.705	9	0.947	0.743	0.247	59.278	58.80	—	14.90	14.00	12.20
	E + H + D + A	0.695	11	0.987	0.826	0.125	198.216	45.10	34.00	—	12.60	8.20
	E + H + S + A	0.74	9	0.984	0.827	0.135	206.773	44.80	29.70	13.60	—	11.90
	E + H + S + D	0.703	9	0.972	0.698	0.178	117.713	48.70	29.70	14.30	7.30	—
	E + H + S + D + A	0.734	9	0.985	0.891	0.132	215.609	41.40	27.60	12.30	11.20	7.50

$q^{2}$: cross-validated correlation coefficient; *ONC*: optimal number of components; $R^{2}$: non-cross-validated correlation coefficient; $r_{\mathrm{pred}}^{2}$: predictive correlation coefficient; *SEE*: standard error of estimate; *F*: F-statistic values; E: electrostatic fields; H: hydrophobic fields; S: steric fields; D: hydrogen-bond donor fields; A: hydrogen-bond acceptor fields.

**Table 2:** External validation parameters of the CoMFA and CoMSIA models.

Statistics	CoMFA (E + S)		CoMSIA (E + H + S + D + A)	
	Training Set	Test Set	Training Set	Test Set
*RMSE*	0.160		0.155	
*MAE*	0.124		0.108	
$r^{2}$	0.9834	0.8764	0.9848	0.8657
$r_{0}^{2}$	0.9830	0.8454	0.9848	0.8613
$r_{0}^{\prime 2}$	0.9831	0.8750	0.9845	0.8144
*k*	1.0003	0.8408	1.0000	1.0848
*k’*	0.9831	0.9982	0.9997	0.9917
$r_{m}^{2}$	0.9637	0.7221	0.9848	0.8083
$r_{m}^{/2}$	0.9664	0.8436	0.9677	0.6696
Δ$r_{m}^{2}$	0.0026	0.1215	0.0171	0.1387
$\bar{r_{m}^{2}}$	0.9690	0.7829	0.9763	0.7389

*RMSE*: root mean square error; *MAE*: mean absolute error.

**Table 3:**
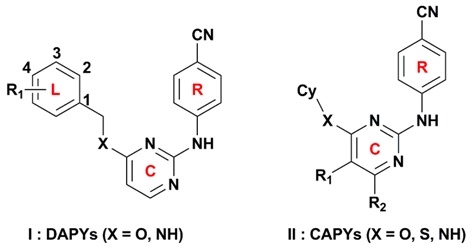
Chemical structures of the selected DAPYs and CAPYs and their actual and predicted pEC_50_ values.

No.	Type	X	R_1_	R_2_	Cy	Actual pEC_50_	CoMFA		CoMSIA	
							Predicted	Residuals	Predicted	Residuals
1	I	O	2-Cl	—	—	7.469	7.318	−0.151	7.355	−0.114
2	I	O	3-Cl	—	—	7.310	7.161	−0.149	7.384	0.074
3	I	O	4-Cl	—	—	6.076	6.172	0.096	5.769	−0.307
4	I	O	2-Br	—	—	7.444	7.283	−0.161	7.442	−0.002
5 *	I	O	3-Br	—	—	7.319	7.196	−0.123	7.277	−0.042
6	I	O	4-Br	—	—	5.328	5.727	0.399	5.733	0.405
7 *	I	O	2-F	—	—	8.237	8.454	0.217	7.750	−0.487
8	I	O	3-F	—	—	7.854	7.737	−0.117	7.848	−0.006
9	I	O	4-F	—	—	6.036	5.964	−0.072	5.927	−0.109
10	I	O	2-CH_3_	—	—	6.796	6.974	0.178	6.831	0.035
11 *	I	O	3-CH_3_	—	—	8.056	7.834	−0.222	8.124	0.068
12	I	O	4-CH_3_	—	—	6.796	6.776	−0.020	6.811	0.015
13	I	O	2-OCH_3_	—	—	7.569	7.551	−0.018	7.488	−0.081
14	I	O	3-OCH_3_	—	—	7.602	7.654	0.052	7.587	−0.015
15	I	O	4-OCH_3_	—	—	6.000	6.080	0.080	6.061	0.061
16	I	O	2-CF_3_	—	—	5.770	5.870	0.100	5.644	−0.126
17	I	O	3-CF_3_	—	—	6.215	6.197	−0.018	6.281	0.066
18	I	O	3,5-diMeO	—	—	8.051	8.086	0.035	8.080	0.029
19	I	O	5-F-2-Br	—	—	7.409	7.411	0.002	7.404	−0.005
20 *	I	O	2-F-4-Br	—	—	6.658	6.841	0.183	6.465	−0.193
21	I	O	2-CN	—	—	7.854	8.000	0.146	7.872	0.018
22	I	O	3-CN	—	—	6.745	6.805	0.060	6.700	−0.045
23	I	O	4-CN	—	—	5.854	5.691	−0.163	5.829	−0.025
24 *	I	O	H	—	—	7.658	7.887	0.229	7.485	−0.173
25	I	O	1-NaPh	—	—	7.824	7.901	0.077	7.804	−0.020
26	I	O	2-NaPh	—	—	6.770	6.726	−0.044	6.772	0.002
27 *	I	NH	H	—	—	8.276	8.139	−0.137	8.036	−0.240
28	I	NH	2-Cl	—	—	7.827	7.956	0.129	7.973	0.146
29	I	NH	3-Cl	—	—	7.924	7.707	−0.217	7.935	0.011
30	I	NH	4-Cl	—	—	7.322	7.106	−0.216	7.216	−0.106
31 *	I	NH	2-Br	—	—	7.376	7.694	0.318	7.026	−0.350
32	I	NH	3-Br	—	—	7.693	7.757	0.064	7.628	−0.065
33	I	NH	4-Br	—	—	7.405	7.389	−0.016	7.223	−0.182
34	I	NH	3-F	—	—	8.357	8.271	−0.086	8.362	0.005
35	I	NH	4-F	—	—	7.143	7.099	−0.044	7.363	0.220
36	I	NH	4-CH_3_	—	—	8.086	8.047	−0.039	8.146	0.060
37 *	I	NH	2-OCH_3_	—	—	7.058	7.270	0.212	7.230	0.172
38	I	NH	3-OCH_3_	—	—	8.347	8.338	−0.009	8.355	0.008
39 *	I	NH	4-OCH_3_	—	—	7.406	7.685	0.279	7.363	−0.043
40	I	NH	2-CF_3_	—	—	5.976	5.771	−0.205	6.101	0.125
41	I	NH	3-CF_3_	—	—	6.687	6.744	0.057	6.615	−0.072
42	I	NH	4-CF_3_	—	—	6.895	6.973	0.078	6.983	0.088
43	I	NH	3,4-diMe	—	—	8.523	8.640	0.117	8.408	−0.115
44	I	NH	2,4-diF	—	—	7.772	7.854	0.082	7.798	0.026
45 *	II	O	H	H	cyclohexyl	5.936	5.775	−0.161	6.130	0.194
46	II	O	H	H	cyclopentyl	5.173	5.140	−0.033	5.297	0.124
47	II	O	H	CH_3_	cyclohexyl	5.188	5.162	−0.026	5.178	−0.010
48	II	NH	H	H	cyclohexyl	6.018	6.046	0.028	5.903	−0.115
49	II	NH	H	H	cyclopentyl	5.801	5.808	0.007	5.746	−0.055
50 *	II	S	H	H	cyclohexyl	7.260	6.874	−0.386	7.371	0.111
51	II	S	H	CH_3_	cyclohexyl	6.367	6.380	0.013	6.425	0.058
52 *	II	S	CH_3_	H	cyclohexyl	7.018	6.633	−0.385	7.399	0.381

* represents test set molecules.

**Table 4:** Distances and angles of hydrogen bonds formed between 3MEC and the docked compounds (**43** and **46**).

Receptor	Ligand	Hydrogen-Bond Receptor	Hydrogen-Bond Donor	Distance (Å)	Angle (°)
3MEC	43	–O (Lys101 –C=O)	–N (–NH)	1.725	151.02
		–N (pyrimidine)	–N (Lys101 –NH_2_)	2.302	168.45
	46	–O (Lys101 –C=O)	–N (–NH)	1.843	157.47
		–N (pyrimidine)	–N (Lys101 –NH_2_)	2.477	163.30

**Table 5:**
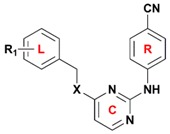
Chemical structure of newly designed DAPYs and their predicted pEC_50_ values based on the CoMFA and CoMSIA models.

No.	R_1_	X	Predicted pEC_50_	
			CoMFA	CoMSIA
53	4-CH_2_CH_3_	NH	8.655	9.508
54	4-CH(CH_3_)_2_	NH	8.765	10.212
55	4-C(CH_3_)_3_	NH	8.276	8.959
56	4-NH_2_	NH	8.695	9.050
57	3-CN	NH	8.334	8.584
58	3-NO_2_	NH	8.306	8.281
59	3-OOCCH_3_	NH	8.227	8.873
60	3-OH	NH	9.020	9.026
61	2-OH	NH	7.845	8.690
62	2-F, 4-CH_3_	NH	8.675	9.388

## Abbreviations

DAPY	diarylpyrimidine
NNRTI	nonnucleoside reverse transcriptase inhibitor
AIDS	acquired immunodeficiency syndrome
3D-QSAR	three-dimensional quantitative structure-activity relationship
CoMFA	comparative molecular field analysis
CoMSIA	comparative molecular similarity indices analysis
HIV	human immunodeficiency virus
HAART	highly active antiretroviral therapy
RT	reverse transcriptase
HIV-1	HIV type 1
WT	wild-type
CAPY	cycloalkyl arylpyrimidine
$q^{2}$	cross-validated correlation coefficient
ONC	optimal number of components
$R^{2}$	non-cross-validated correlation coefficient
$r_{\mathrm{pred}}^{2}$	predictive correlation coefficient
SEE	standard error of estimate
F	F-statistic values
RMSE	root mean square error
MAE	mean absolute error
GALAHAD	Genetic Algorithm with Linear Assignment of Hypermolecular Alignment of Datasets
SE	strain energy
SO	steric overlap
PhS	pharmacophoric similarity
DA	hydrogen-bond donor atom
AA	hydrogen-bond acceptor atom
HY	hydrophobe
RMSD	root-mean-square deviation
LOO	leave one out
SDEP	standard deviation error of predictions
PRESS	predicted residuals sum of squares
